# Fostering the next generation of bone marrow adiposity researchers: the 3rd International Bone Marrow Adiposity Society Summer School 2025 report

**DOI:** 10.1242/bio.062396

**Published:** 2026-03-04

**Authors:** Drenka Trivanović, Tânia Amorim, Adriana Roque, Souad Daamouch, Young-Eun Park, Tiange Feng, Maxime Bedez, Izabela Podgorski

**Affiliations:** ^1^ Group for Hematology and Stem Cells, Institute for Medical Research, University of Belgrade, 11000 Belgrade, Serbia; ^2^ University of Pittsburgh School of Medicine, Division of Endocrinology and Metabolism, 15206 Pittsburgh, USA; ^3^ Hospital da Universidade de Coimbra, Unidade Local de Saúde de Coimbra, Clinical Hematology Department, 3004-561 Coimbra, Portugal; ^4^ Faculty of Medicine, University of Coimbra, 3004-561 Coimbra, Portugal; ^5^ Department of Clinical Research, University of Southern Denmark, DK-5000 Odense, Denmark; ^6^ Molecular Endocrinology & Stem Cell Research Unit, Department of Endocrinology, Odense University Hospital, DK-5000 Odense, Denmark; ^7^ Nuffield Department of Surgical Sciences, University of Oxford, Oxford OX3 7LD, UK; ^8^ MaineHealth Institute for Research, Center for Molecular Medicine, 81 Research Drive, Scarborough 04074, Maine, USA; ^9^ MABLab – Marrow Adiposity & Bone Laboratory, Faculté de Chirurgie Dentaire de Lille, Univ. Lille, CHU Lille, Univ. Littoral Côte d'Opale, ULR 4490, Pl. de Verdun, Lille, France; ^10^ Department of Pharmacology, Wayne State University School of Medicine, Detroit, MI 48201, USA

**Keywords:** Bone marrow adipose tissue, Bone marrow adipocyte, Bone, Hematopoiesis, Single cell, Young researcher

## Abstract

The 3rd International Bone Marrow Adiposity Society (BMAS) Summer School 2025 gathered young basic and clinical researchers to advance their knowledge of bone marrow adipose tissue (BMAT). The virtual BMAS Summer School 2025 consisted of panels featuring keynote speeches, workshops, career development lectures, and panel discussions. A total of 19 speakers and 50 attendees participated in the event. The program included sessions on BMAT characterization, mechanosensitivity, metabolism, relationships with the skeletal and hematopoietic systems, and nutritional interventions. Workshop sessions provided knowledge on *ex vivo* culture systems, single-cell and nucleus resolution techniques, as well as advanced and clinical imaging. The virtual format enabled accessible international participation, creating a structured environment for young researchers to share research, develop skills, and build new connections.

## Teaser

**Summary:** The BMAS Summer School 2025 program spanned three days, covering BMAT from molecular biology to pathology via expert lectures, workshops, and interactive sessions. It educated young investigators from all over the world in cutting-edge techniques, BMAT's role in niche/disease contexts, and BMAT-targeted therapeutic strategies.

## Introduction

The International Bone Marrow Adiposity Society (BMAS) was established to advance our understanding of the role of bone marrow adipose tissue (BMAT) in health and disease. The BMAS Summer School 2025 is a 3-day online event dedicated to early-stage scientists in the field of BMAT. President of the BMAS, Dr. Bram van der Eerden (University Medical Centre Rotterdam, The Netherlands) introduced the BMAS Summer School 2025 as the 11th event in the organization of BMAS and 4th in virtual form (Amorim et al., 2024; Labella et al., 2022; Scheller et al., 2021). The BMAS Summer School 2025 featured a blend of lectures by leading experts, interactive workshops, and career development sessions. Particularly, the program provided state-of-the art knowledge on bone marrow (BM) compartments, imaging, BMAT features and scientific writing and leadership skills development. The goal of this report is to describe the structure and outcomes of the BMAS Summer School 2025.

## BMAS Summer School 2025 – core topics overview

### Specialization of BM cells and 3D imaging techniques

**Fig. 1.**
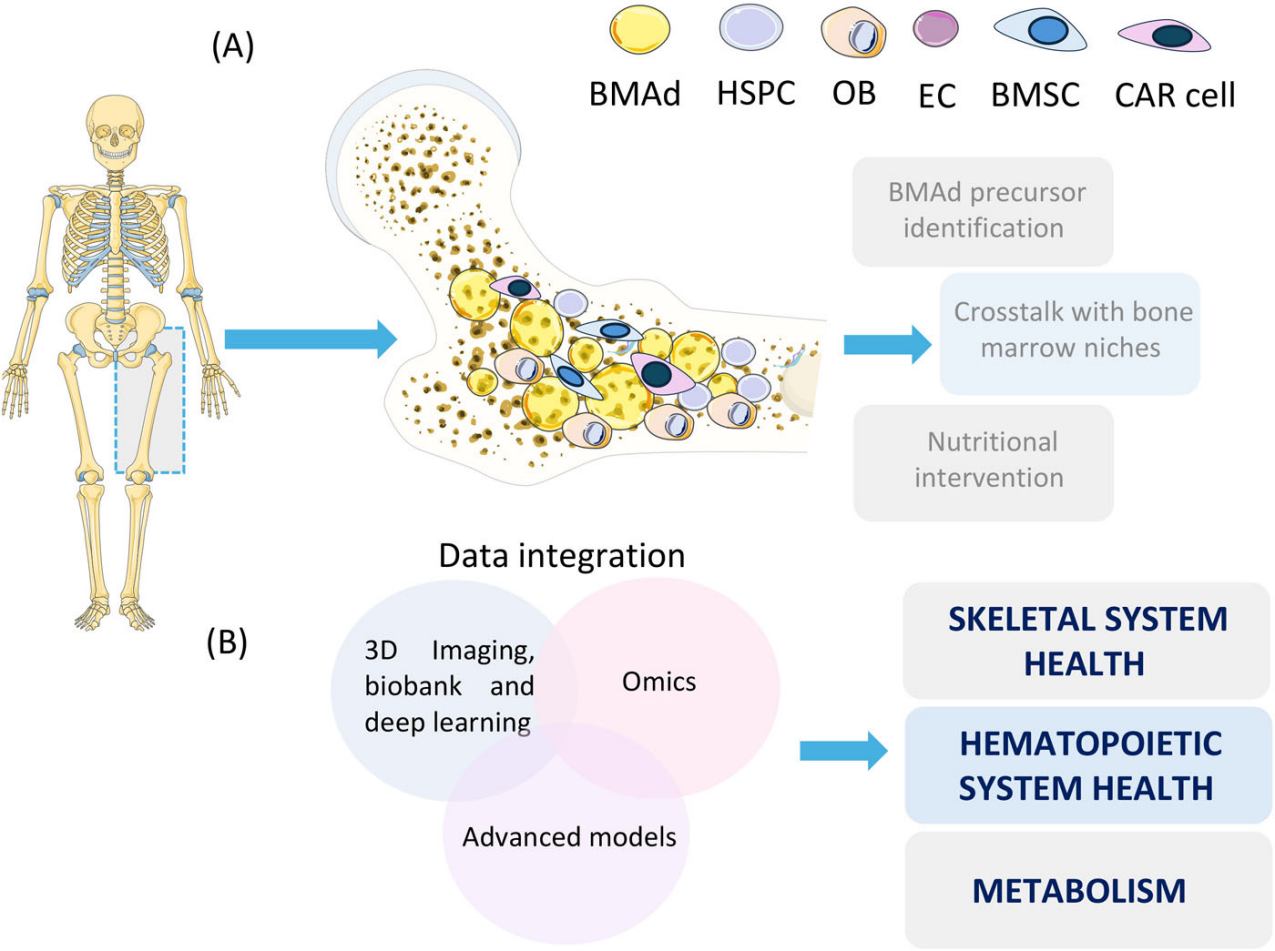
**Landscape of BMAT in current studies dedicated to BMAd precursor identification, defining niche-specific interactions and targeting BMAT to combat diseases.** Overview of (A) discussed bone marrow compartments, BMAd identification and interventions to modify BMAT; (B) data integration including advanced (clinical) imaging, sc-RNA and sn-RNA sequencing, and *ex vivo* models to deconvolute BMAT architecture and physiology. BMAd, bone marrow adipocyte; HSPC, hematopoietic stem and progenitor cell; OB, osteoblast; EC, endothelial cell; BMSC, bone marrow stromal cell; CAR cell, CXCL12-abundant reticular cell. Figure created with smart.servier.com.

Dr. Ralf Adams (Max Planck Institute for Molecular Biomedicine, University of Münster, Münster, Germany) gave the first keynote lecture entitled “Specialization of bone marrow cells and microenvironment”. Dr. Adams provided insight into the importance of vasculature for skeletal system homeostasis and BM colonization by hematopoietic stem and progenitor cells (HSPCs) (Sivaraj and Adams, 2016). He emphasized the critical intercellular crosstalk among osteolineage cells, HSPCs, and endothelial cells within the bone marrow (Fig. 1), noting that myelofibrosis and osteosclerosis profoundly disturb this interplay. Adams' group found that the gene for Hippo kinase large tumor suppressor kinase 2 (LATS2) triggers endothelial-to-mesenchymal transition, resulting in increased production of extracellular matrix components and signaling molecules. This shows the endothelium drives BM fibrosis, enhancing understanding of myelofibrotic and osteosclerotic diseases (Sivaraj et al., 2024). Dr. Adams presented his ongoing research on the heterogeneity of BM cells across skeletal sites, which reflects different BM composition in terms of vasculature, HSPCs and BM adipocytes (BMAds) at different developmental stages (Koh et al., 2024). Altogether, showcasing outstanding results in the field, Dr. Adams introduced young investigators to the important aspects of BM research.

Dr. César Nombela-Arrieta (University of Zurich, Zurich, Switzerland) gave a workshop talk entitled “Cutting-edge techniques for 3D imaging of BM niches”. Dr. Nombela-Arrieta presented investigations of the architecture of BM tissues, non-hematopoietic stromal cells formed specialized niches (Baccin et al., 2020) and networks that maintain HSPCs. Dr. Nombela-Arietta showed how multiscale quantitative 3D imaging can be optimized for BM-wide view and using tools such as optical segmentation to boost the quality BM resolution. He presented how his group established a pipeline for 3D deep tissue imaging of thick slices of non-decalcified bones (Mun and Nombela-Arrieta, 2021). Thus, mouse bones can be prepared for confocal imaging, quantitative imaging, spatial analyses and deep learning of non-hematopoietic cells such are CXCL12-abundant reticular cells within BM, which cannot be precisely characterized by flow cytometry (Gomariz et al., 2020, 2021). Dr. Nombela-Arrieta demonstrated how these methods can be utilized for dysregulated BM, ending with recent 3D imaging of human BM reticular and adipogenic regions. Collectively, these lectures provided fundamental knowledge on BM physiology and most contemporary imaging strategy for accurate estimation of cells and BMAT within BM niche. Attendees were introduced to the fundamental structure of the BM, opening new perspectives for designing and performing their research projects more effectively.

### Insights into BMAT mechanosensitivity and metabolism

In the next session, we discussed a relatively new field of BMAT mechanosensitivity, as well as important metabolic features of BMAT and adipocytes. Dr. Glenn Niebur (University of Notre Dame, USA) presented a lecture on “BMAT mechanosensitivity”, highlighting the importance of looking at bone and marrow as parts of an integrated mechanical system. His lecture demonstrated that bone deformation creates pressure gradients and shear stresses within the trabecular marrow space, providing new insights into how mechanical loading affects bone physiology (Curtis et al., 2020). Shear stress in the BM influences protein phosphorylation and gene expression, affecting bone formation, while BMAT changes shear forces on BM stromal cells (BMSCs), potentially altering their differentiation. These findings support the idea that bone and marrow function as a mechanically connected system, making BM mechanobiology a key regulator of bone homeostasis and a potential target for osteoporosis research and therapy.

In a lecture entitled “Metabolic functions of BMAT: from fundamental biology to human health”, Dr. William Cawthorn (University of Edinburgh, UK) shared new insights into the metabolic roles of BMAT. He showed that BMAT is a unique and active fat depot involved in lipid and glucose metabolism (Suchacki et al., 2016). Dr. Cawthorn presented preclinical and clinical evidence from his group showing that BMAT contributes to systemic energy homeostasis through lipid storage and mobilization, influencing skeletal and metabolic health (Suchacki et al., 2020). He also discussed UK Biobank imaging analyses that link BMAT quantity (Fig. 1) and composition with insulin sensitivity and bone density (Xu et al., 2025). Collectively, this large-scale resource offered unique opportunities to study BMAT in humans, to understand its physiological and pathological roles, and to explore the genetic and environmental factors that influence BMAT.

Dr. Shingo Kajimura (Harvard University, USA) gave a talk about “Mitochondrial regulation and adipocyte homeostasis”, describing the importance of temperature regulation and heat generation in biological processes. He explored the mechanisms of thermogenesis, including both classical UCP1-dependent pathways (mainly in brown adipose tissue) and UCP1-independent pathways such as the calcium cycling pathway (Cohen and Kajimura, 2021). These UCP1-independent processes contribute to whole-body energy expenditure and may influence insulin sensitivity and metabolic health. By comparing data from different tissues, species, and adipose depots, he showed how non-shivering thermogenesis is evolutionarily conserved and could be relevant to BMAT, suggesting that these pathways might reveal new targets for metabolic and skeletal disorders. These metabolic aspects of BMAT helped young scientists to understand how BMAT metabolism interacts with skeletal system and systemic stimuli. This knowledge will expand their perspective on BMAT's involvement in skeletal health and systemic metabolic regulation.

### Determining BMAT at single cell and nucleus resolution

Dr. Ling Qin's (University of Pennsylvania, USA) presentation detailed her lab's work using single-cell RNA-sequencing (scRNA-seq) to unravel the complex heterogeneity of mesenchymal lineage cells within the BM (Fig. 1). Her research identified distinct subpopulations, including early mesenchymal progenitors (EMPs), and a key population they termed marrow adipogenic lineage precursors (MALPs). The research focused heavily on the functional roles of MALPs. Using an *Adipoq*-Cre model to specifically trace these cells, which express high levels of *Pparγ*, they discovered that *Adipoq* expression initiates in MALPs before lipid accumulation, marking them as early-stage adipogenic cells. Conversely, MALPs were also identified as a major source of osteoclast regulators RANKL and CSF1, revealing their critical role in bone resorption and remodeling, particularly in contexts like OVX-induced bone loss. Furthermore, *Csf1* deficiency in MALPs resulted in increased bone mass and fewer BM macrophages and HSPCs, highlighting their importance in the BM niche. The team also found that MALPs are crucial for BM repair after radiation, where they acquire a myofibroblast gene profile to aid in recovery (Zhong et al., 2020). Translating these findings to humans, scRNA-seq of human BM identified analogous cell types, including Adipo-BMSCs and Thy1^+^ BMSCs, which are major sources of hematopoietic supporting factors (Zhong et al., 2020). To conclude, Dr. Qin introduced the lab's ongoing work using advanced spatial imaging (co-detection by indexing) to map the precise locations of these distinct BMSC subsets within the BM niche.

Dr. Ryan Chai's (Garvan Institute of Medical Research, Australia) presentation, "Bone at single cell resolution," provided a comprehensive overview of advanced transcriptomic technologies, with a particular focus on the challenges and solutions for studying BMAds. The classic method, scRNA-seq, was highlighted as a robust and widely adopted technique, powerful for characterizing diverse cell populations of BM, including hematopoietic, endothelial, and mesenchymal lineages. Dr. Chai showcased its utility in mapping the differentiation trajectory from BMSCs to mature osteocytes. However, he emphasized a critical limitation: the enzymatic and mechanical dissociation required for scRNA-seq often destroys fragile, lipid-laden cells like BMAds, leading to their underrepresentation in datasets. Moreover, he introduced single-nucleus RNA-sequencing (snRNA-seq) as a superior alternative for BMAd analysis (Ziegenhain et al., 2017; Chai, 2022). By isolating nuclei directly from frozen tissue, snRNA-seq bypasses the harsh cell dissociation step, preserving the integrity of fragile cell types. Finally, Dr. Chai presented spatial transcriptomics as the most promising frontier for BMAd research, which allows for gene expression analysis directly within the context of an intact tissue section, preserving the crucial spatial information of where cells reside in the niche. The talk concluded that while each technology has its strengths, the future lies in a multi-modal approach, integrating the cellular depth of sc/snRNA-seq with the spatial transcriptomics to build an atlas of the BM microenvironment.

### Bone marrow adipocytes: implications in skeletal and hematopoietic system health

Dr. Clifford Rosen's (MaineHealth Institute for Research, USA) lecture, “Bone marrow adiposity and skeletal health”, addressed the intricate and often paradoxical role of BMAT in regulating the skeleton (Fig. 1). His presentation established that while BMAT is known to increase with age and in conditions like osteoporosis, its function is far from passive. Dr. Rosen highlighted a paradox: both high-fat diet (HFD) and calorie restriction (CR) increase BMAT, but while HFD harms bone, CR causes cortical bone loss yet preserves or improves trabecular bone. This suggests BMAT's role depends on metabolic context. A primary hypothesis explored was that BMAT serves as a local energy source for bone cells during periods of nutritional stress. This was assessed using genetic models with impaired lipolysis in BM adipocytes (BMAd-Pnpla2^−^/^−^ mice), in which the protective effect of CR on trabecular bone was lost, leading to bone loss (Li et al., 2022b). Dr. Rosen highlighted that BMAT supplies fatty acids through lipolysis to maintain skeletal integrity during an energy deficit, thus functioning as a compensatory and supportive depot. Conversely, the presentation also detailed BMAT's role as a negative regulator of bone formation. In summary, Dr. Rosen portrayed BMAT as a dynamically adaptive tissue with a dual nature, that can act to suppress bone formation under basal conditions but transforms into a vital local fuel source that actively supports bone maintenance under stress conditions.

Two outstanding talks at this year's summer school highlighted the emerging role of BMAds in regulating the hematopoietic niche in health and disease. Dr. Ziru Li (MaineHeath Institute for Research, USA) focused on dissecting the role of BMAds in hematopoietic regulation. She noted that defining BMAd function has been challenging due to the lack of BMAd-specific models with full penetrance. To address this, Dr. Li and the MacDougald laboratory generated a BMAd-specific Cre mouse model based on endogenous *Osterix* and *Adipoq* expression and selectively deleted the lipolytic gene ATGL (Pnpla2) in BMAds (*BMAd-Pnpla2–/–* mice) (Li et al., 2022b). These mice exhibited impaired BMAd lipolysis and an increased baseline size and number of BMAds. Because a single BMAd can physically associate with nearly 100 hematopoietic cells, the group hypothesized that BMAds provide metabolic support for hematopoiesis. This study suggested that BMAd-derived lipids are required for myeloid lineage regeneration when circulating energy supplies are limited. In complementary studies, BMAd depletion via diphtheria toxin A expression resulted in significant reductions in HSPCs (Li et al., 2022a). Dr. Li also highlighted context-dependent roles of BMAds in inflammatory stress responses.

Dr. Claire Edwards (University of Oxford, UK) gave an overview of the current understanding and recent advancements in BMAd contribution to multiple myeloma (MM) development and progression. Dr. Edwards highlighted prior evidence linking HFD-induced obesity with MM progression (Lwin et al., 2015). She presented previously published data demonstrating that BMAT was increased in the initial stages of disease and, as MM advances, it became concentrated at the tumor–bone interface (Morris et al., 2020). Dr. Edwards described several mechanisms through which BMAds promote MM progression, including MM-driven loss of adiponectin (Fowler et al., 2011), metabolic crosstalk between BMAds and MM cells (Roman-Trufero et al., 2022) and BMAd acquisition of a senescence-associated secretory phenotype (Fairfield et al., 2021). Notably, unpublished findings from Edwards' group revealed the contribution of obesity and ageing, both known to increase BMAT, in progression from monoclonal gammopathy of undetermined significance (MGUS) to MM. Finally, Dr. Edwards presented preliminary findings suggesting that BMAds may reduce MM dormancy *in vitro* and *in vivo*, and highlighted the need for further investigation. Altogether, these lectures emphasized important and dual roles of BMAT in skeletal regeneration as well as malignancies, enabling young researchers to recognize modulatory features of BMAT.

### Nutritional interventions for the regulation of BMAT

Dr. Tim J. Schulz (German Institute of Human Nutrition, Germany) presented a lecture, “Nutritional interventions in regulation of bone marrow adiposity”, where he explained the influence of nutrition and ageing on BM adiposity and its impact on bone integrity. BMAT can comprise up to 20% of total body fat, increasing with age, injury or irradiation, obesity, diabetes, and even CR (Tavassoli, 1976; Scheller et al., 2015). Schulz's group demonstrated that both energy surplus and energy depletion promote the accumulation of BMAds. Dietary interventions in mice revealed that short-term HFD enhanced the commitment of BMSCs to adipogenesis (Ambrosi et al., 2017), whereas high-sucrose diets had minimal effects on bone quality. Transcriptomic analyses linked these improvements to enhanced mitochondrial function and increased adiponectin secretion, suggesting a shift towards metabolically ‘healthy’ BMAT (Rinne et al., 2024). He explained that blocking dipeptidyl peptidase-4 (DPP4) restored the bone healing impaired by BMAT and may offer a new treatment route (Ambrosi et al., 2017). Collectively, Dr. Schulz's findings illustrate how nutritional and metabolic interventions can reshape BMAT quality (Fig. 1) and potentially mitigate age-related skeletal decline.

### *Ex vivo* models and clinical imaging of BMAT

The complexity of BMAT requires experimental models that capture its unique interactions within the BM microenvironment while remaining applicable to clinical settings. Dr. Michaela Reagan (MaineHealth Institute for Research, USA) emphasized that conventional 2D adipocyte cultures only partially reflect BM complexity. Tissue-engineered BMAT offers a more physiologically relevant model of human BMAT. To achieve this, her group developed *ex vivo* systems using silk fibrin-based 3D scaffolds co-cultured with stromal and tumor cells (Fairfield et al., 2019). These biomimetic silk matrices support the long-term maintenance of BMAds, enabling investigation of their metabolic and paracrine interactions with multiple myeloma cells, which can hijack BMAds and neighboring stromal populations (Dadwal et al., 2016). Such models revealed the active role of BMAT in hematologic malignancies, providing insights into fatty acid exchange and chemoresistance. These approaches enable the interrogation of BMAd-specific contributions, bridging the gap between *in vitro* studies and *in vivo* complexity (Fairfield et al., 2023). These 3D models hold great translational potential to accelerate BMAT research.

Complementing these findings, Dr. Dimitrios Karampinos (Technical University of Munich, Germany) outlined advanced MRI and chemical shift–based imaging methods for assessing BMAT in humans (Yeung et al., 2005). Building on these advances, his research has established reliable techniques to quantify bone marrow fat fraction, lipid composition, and spatial distribution (Honecker et al., 2022). He emphasized their translational relevance across metabolic, osteoporotic, and oncologic contexts. Advances in imaging resolution and the capacity to differentiate saturated versus unsaturated lipid fractions provide a window into BMAT physiology *in vivo*. Recent UK Biobank studies, including the OPTIMAT project, extend these findings through automated MRI analyses linking BMAT to genetic and disease traits. These population-scale insights, derived from UK Biobank imaging (Xu et al., 2025), underscore how advances such as OPTIMAT are transforming BMAT quantification into a powerful epidemiological and clinical biomarker, bridging basic imaging research with population health applications. Together, presented 3D *ex vivo* and clinical-imaging techniques illustrated approaches toward integrating molecular- and population-level understanding of BMAT (Fig. 1), enabling translational advance of BMAT research. This should encourage basic and clinical BMAT researchers to intensify their project work, increasing their network with experts from different fields.

## Career advancement

Navigating professional and career development in biomedicine is essential for sustaining long-term progress and innovation within the field. Speakers at the BMAS Summer School 2025 shared valuable insights on grant writing, transitioning between academia and industry, and establishing successful independent research groups, contributing to building enduring and rewarding scientific careers.

Effective grant writing remains a cornerstone of scientific advancement. Dr. Ormond MacDougald (University of Michigan, USA) emphasized the importance of clear communication, encouraging researchers to guide reviewers who may not be familiar with their specific area of expertise. Strong preliminary data and logical experimental design were presented as key elements that can make proposals more compelling. Dr. MacDougald emphasized that viewing a grant through the eyes of a reviewer can significantly enhance its overall quality and increase success rates.

Establishing and leading a research group represents another crucial aspect of professional development. Dr. Erica Scheller (Washington University School of Medicine, USA) discussed the early stages of creating a laboratory, encouraging self-reflection and careful planning. Tools such as the ‘Cantril Ladder’ can help researchers evaluate their satisfaction, set meaningful goals and allocate time strategically to nurture both personal and professional growth.

Career sustainability also involves making thoughtful transitions between academia and industry. Dr. Barna Gal (CompagOs, Switzerland) shared his personal journey from orthopaedic surgery to entrepreneurship, underlining the importance of aligning career choices with personal values and motivations. Dr. Davide K. Ebri (UCB Pharma, UK) noted that academia is driven by curiosity, while industry focuses on therapeutic targets. Both speakers highlighted adaptability and the ability to learn from setbacks as essential skills for maintaining enthusiasm and balance throughout a career.

## Integrative sustainability achievements of the BMAS Summer School 2025

**Fig. 2.**
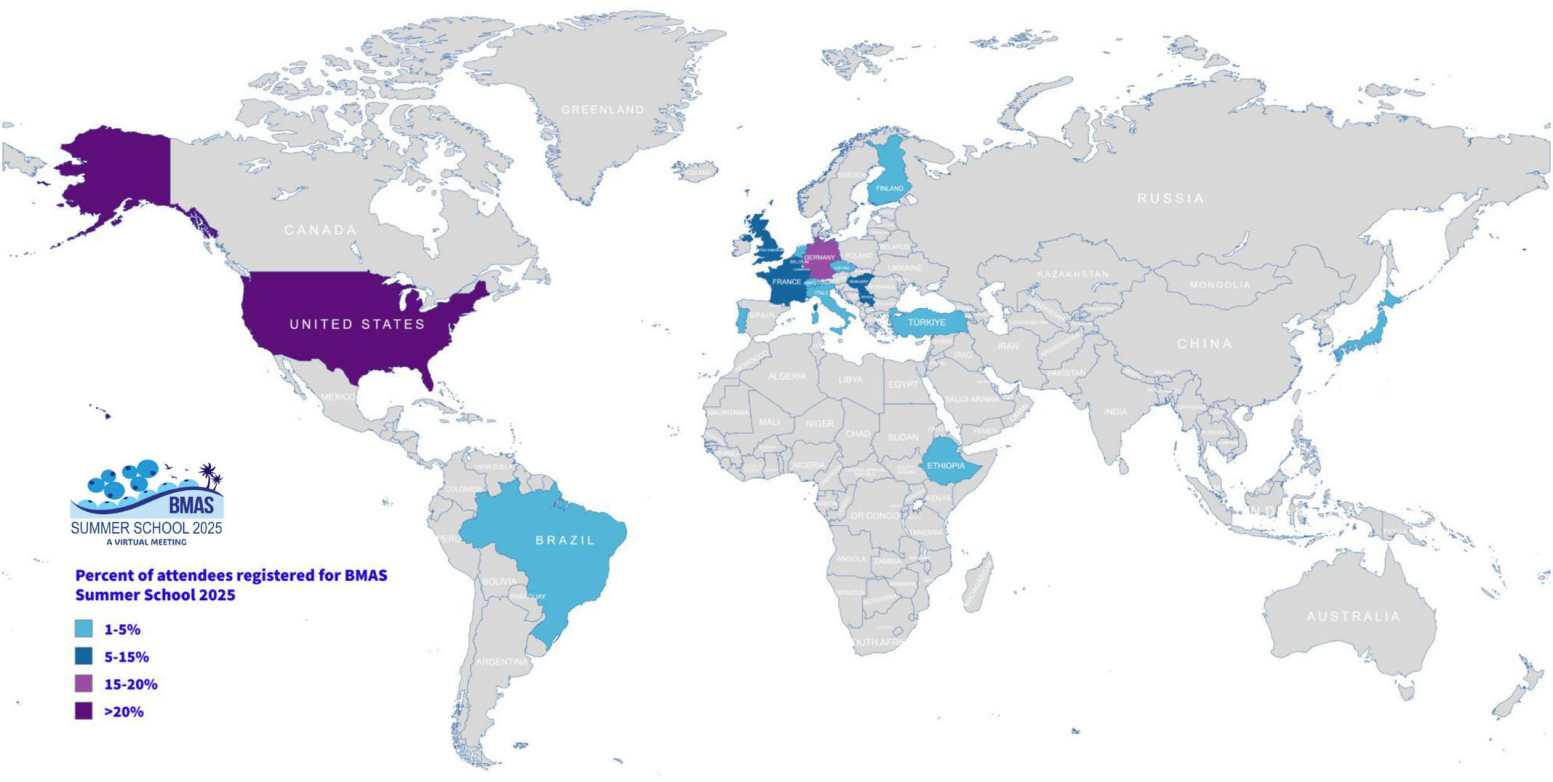
**The international reach of the BMAS Summer School 2025 with indicated percentages of participants from each country.** Number of participants per country: Belgium (3), Brazil (1), Czech Republic (1), Ethiopia (1), Finland (1), France (4), Germany (8), Hungary (3), Italy (1), Japan (1), Netherlands (2), Portugal (1), Serbia (3), Switzerland (2), Turkey (1), UK (4), USA (10). Map created using MapChart.net.

Recognition of established and young investigators encourages sustained engagement, excellence, and collaboration. Award honored Dr. William Cawthorn (UK) and Dr. Julien Paccou (France) as best BMAS basic and clinical scientists. Veronika Malkova (Czech Republic) received best abstract; Polona Kalc (Germany) best presentation; Sara Peripinello (Italy) most active participant award. To promote equity, awards supported researchers from low- and middle-income countries (Ethiopia, Serbia, and Brazil), fostering global collaboration. We concluded that clear communication, inclusivity, adaptability, self-reflection and recognition of achievements contribute to sustainable and fulfilling careers in BMAT research. Organized as a virtual event, the 3rd BMAS Summer School, brought together researchers from all over the world (Fig. 2), reducing travel-related costs, fostering equitable participation while minimizing the event's overall environmental footprint.

## Summary

The 2025 BMAS Summer School offered valuable opportunities for early-stage scientists to exchange knowledge and build a more connected BMAT research community over the world. The virtual format of the event enabled broad international participation, creating an accessible environment for early-stage scientists to share research, develop skills, and build new connections. The virtual format of the Summer School proved to be effective, attracting a number of participants. Despite challenges in communication typical of virtual settings, the active engagement and discussions demonstrated that meaningful interaction was well supported. This indicates that virtual Summer Schools can successfully provide flexible and accessible learning opportunities while maintaining solid communication between participants and lecturers.

## References

[BIO062396C1] Ambrosi, T. H., Scialdone, A., Graja, A., Gohlke, S., Jank, A. M., Bocian, C., Woelk, L., Fan, H., Logan, D. W., Schürmann, A. et al. (2017). Adipocyte accumulation in the bone marrow during obesity and aging impairs stem cell-based hematopoietic and bone regeneration. *Cell Stem Cell* 20, 771-784.e6. 10.1016/j.stem.2017.02.009 28330582 PMC5459794

[BIO062396C2] Amorim, T., Trivanovic, D., Benova, A., Li, H., Tencerova, M. and Palmisano, B. (2024). Young minds, deeper insights: a recap of the BMAS Summer School 2023, ranging from basic research to clinical implications of bone marrow adipose tissue. *Biol. Open* 13, bio060263. 10.1242/bio.060263 38288785 PMC10855210

[BIO062396C3] Baccin, C., Al-Sabah, J., Velten, L., Helbling, P. M., Grünschläger, F., Hernández-Malmierca, P., Nombela-Arrieta, C., Steinmetz, L. M., Trumpp, A. and Haas, S. (2020). Combined single-cell and spatial transcriptomics reveal the molecular, cellular and spatial bone marrow niche organization. *Nat. Cell Biol.* 22, 38-48. 10.1038/s41556-019-0439-6 31871321 PMC7610809

[BIO062396C4] Chai, R. C. (2022). Single-Cell RNA sequencing: unravelling the bone one cell at a time. *Curr. Osteoporos Rep.* 20, 356-362. 10.1007/s11914-022-00735-w 35915289 PMC9522837

[BIO062396C5] Cohen, P. and Kajimura, S. (2021). The cellular and functional complexity of thermogenic fat. *Nat. Rev. Mol. Cell Biol.* 22, 393-409. 10.1038/s41580-021-00350-0 33758402 PMC8159882

[BIO062396C6] Curtis, K. J., Oberman, A. G. and Niebur, G. L. (2020). Effects of mechanobiological signaling in bone marrow on skeletal health. *Ann. N. Y. Acad. Sci.* 1460, 11-24. 10.1111/nyas.14232 31508828

[BIO062396C7] Dadwal, U., Falank, C., Fairfield, H., Linehan, S., Rosen, C. J., Kaplan, D. L., Sterling, J. and Reagan, M. R. (2016). Tissue-engineered 3D cancer-in-bone modeling: silk and PUR protocols. *BoneKEy Rep.* 5, 842. 10.1038/bonekey.2016.75 27790370 PMC5070496

[BIO062396C8] Fairfield, H., Falank, C., Farrell, M., Vary, C., Boucher, J. M., Driscoll, H., Liaw, L., Rosen, C. J. and Reagan, M. R. (2019). Development of a 3D bone marrow adipose tissue model. *Bone* 118, 77-88. 10.1016/j.bone.2018.01.023 29366838 PMC6062483

[BIO062396C9] Fairfield, H., Dudakovic, A., Khatib, C. M., Farrell, M., Costa, S., Falank, C., Hinge, M., Murphy, C. S., DeMambro, V., Pettitt, J. A. et al. (2021). Myeloma-modified adipocytes exhibit metabolic dysfunction and a senescence-associated secretory phenotype. *Cancer Res.* 81, 634-647. 10.1158/0008-5472.CAN-20-1088 33218968 PMC7854508

[BIO062396C10] Fairfield, H., Condruti, R., Farrell, M., Di Iorio, R., Gartner, C. A., Vary, C. and Reagan, M. R. (2023). Development and characterization of three cell culture systems to investigate the relationship between primary bone marrow adipocytes and myeloma cells. *Front. Oncol.* 12, 912834. 10.3389/fonc.2022.912834 36713534 PMC9874147

[BIO062396C11] Fowler, J. A., Lwin, S. T., Drake, M. T., Edwards, J. R., Kyle, R. A., Mundy, G. R. and Edwards, C. M. (2011). Host-derived adiponectin is tumor-suppressive and a novel therapeutic target for multiple myeloma and the associated bone disease. *Blood* 118, 5872-5882. 10.1182/blood-2011-01-330407 21908434 PMC3228502

[BIO062396C12] Gomariz, A., Isringhausen, S., Helbling, P. M. and Nombela-Arrieta, C. (2020). Imaging and spatial analysis of hematopoietic stem cell niches. *Ann. N. Y. Acad. Sci.* 1466, 5-16. 10.1111/nyas.14184 31368140

[BIO062396C13] Gomariz, A., Portenier, T., Helbling, P. M., Isringhausen, S., Suessbier, U., Nombela-Arrieta, C. and Goksel, O. (2021). Modality attention and sampling enables deep learning with heterogeneous marker combinations in fluorescence microscopy. *Nat. Mach. Intell.* 3, 799-811. 10.1038/s42256-021-00379-y 34541455 PMC7611676

[BIO062396C14] Honecker, J., Ruschke, S., Seeliger, C., Laber, S., Strobel, S., Pröll, P., Nellaker, C., Lindgren, C. M., Kulozik, U., Ecker, J. et al. (2022). Transcriptome and fatty-acid signatures of adipocyte hypertrophy and its non-invasive MR-based characterization in human adipose tissue. *EBioMedicine* 79, 104020. 10.1016/j.ebiom.2022.104020 35490555 PMC9062743

[BIO062396C15] Koh, B. I., Mohanakrishnan, V., Jeong, H. W., Park, H., Kruse, K., Choi, Y. J., Nieminen-Kelhä, M., Kumar, R., Pereira, R. S., Adams, S. et al. (2024). Adult skull bone marrow is an expanding and resilient haematopoietic reservoir. *Nature* 636, 172-181. 10.1038/s41586-024-08163-9 39537918 PMC11618084

[BIO062396C16] Labella, R., Little-Letsinger, S., Avilkina, V., Sarkis, R., Tencerova, M., Vlug, A. and Palmisano, B. (2022). Next generation bone marrow adiposity researchers: report from the 1st BMAS summer school 2021. *Front. Endocrinol.* 13, 879588. 10.3389/fendo.2022.879588 PMC9043644 35498418

[BIO062396C17] Li, Z., Bagchi, D. P., Zhu, J., Bowers, E., Yu, H., Hardij, J., Mori, H., Granger, K., Skjaerlund, J., Mandair, G. et al. (2022a). Constitutive bone marrow adipocytes suppress local bone formation. *JCI Insight* 7, e160915. 10.1172/jci.insight.160915 36048537 PMC9675472

[BIO062396C18] Li, Z., Bowers, E., Zhu, J., Yu, H., Hardij, J., Bagchi, D. P., Mori, H., Lewis, K. T., Granger, K., Schill, R. L. et al. (2022b). Lipolysis of bone marrow adipocytes is required to fuel bone and the marrow niche during energy deficits. *eLife* 11, e78496. 10.7554/eLife.78496 35731039 PMC9273217

[BIO062396C19] Lwin, S. T., Olechnowicz, S. W., Fowler, J. A. and Edwards, C. M. (2015). Diet-induced obesity promotes a myeloma-like condition in vivo. *Leukemia* 29, 507-510. 10.1038/leu.2014.295 25287992

[BIO062396C20] Morris, E. V., Suchacki, K. J., Hocking, J., Cartwright, R., Sowman, A., Gamez, B., Lea, R., Drake, M. T., Cawthorn, W. P. and Edwards, C. M. (2020). Myeloma cells down-regulate adiponectin in bone marrow adipocytes Via TNF-Alpha. *J. Bone Miner. Res.* 35, 942-955. 10.1002/jbmr.3951 31886918 PMC9328417

[BIO062396C21] Mun, Y. and Nombela-Arrieta, C. (2021). 3D Microscopy of murine bone marrow hematopoietic tissues. *Methods Mol. Biol.* 2308, 127-138. 10.1007/978-1-0716-1425-9_11 34057720

[BIO062396C22] Rinne, C., Soultoukis, G. A., Oveisi, M., Leer, M., Schmidt-Bleek, O., Burkhardt, L. M., Bucher, C. H., Moussa, E. A., Makhlouf, M., Duda, G. N. et al. (2024). Caloric restriction reduces trabecular bone loss during aging and improves bone marrow adipocyte endocrine function in male mice. *Front. Endocrinol. (Lausanne)* 15, 1394263. 10.3389/fendo.2024.1394263 38904042 PMC11188307

[BIO062396C23] Roman-Trufero, M., Auner, H. W. and Edwards, C. M. (2022). Multiple myeloma metabolism - a treasure trove of therapeutic targets? *Front. Immunol.* 13, 897862. 10.3389/fimmu.2022.897862 36072593 PMC9441940

[BIO062396C24] Scheller, E. L., Doucette, C. R., Learman, B. S., Cawthorn, W. P., Khandaker, S., Schell, B., Wu, B., Ding, S. Y., Bredella, M. A., Fazeli, P. K. et al. (2015). Region-specific variation in the properties of skeletal adipocytes reveals regulated and constitutive marrow adipose tissues. *Nat. Commun.* 6, 7808. 10.1038/ncomms8808 26245716 PMC4530473

[BIO062396C25] Scheller, E. L., McGee-Lawrence, M. E. and Lecka-Czernik, B. (2021). Report From the 6th international meeting on bone marrow adiposity (BMA2020). *Front. Endocrinol.* 12, 712088. 10.3389/fendo.2021.712088 PMC8323480 34335478

[BIO062396C26] Sivaraj, K. K. and Adams, R. H. (2016). Blood vessel formation and function in bone. *Development (Cambridge, England)* 143, 2706-2715. 10.1242/dev.136861 27486231

[BIO062396C27] Sivaraj, K. K., Majev, P. G., Dharmalingam, B., Schröder, S., Banjanin, B., Stehling, M., Zeuschner, D., Nordheim, A., Schneider, R. K. and Adams, R. H. (2024). Endothelial LATS2 is a suppressor of bone marrow fibrosis. *Nat. Cardiovasc. Res.* 3, 951-969. 10.1038/s44161-024-00508-x 39155965 PMC11324521

[BIO062396C28] Suchacki, K. J., Cawthorn, W. P. and Rosen, C. J. (2016). Bone marrow adipose tissue: formation, function and regulation. *Curr. Opin. Pharmacol.* 28, 50-56. 10.1016/j.coph.2016.03.001 27022859 PMC5351553

[BIO062396C29] Suchacki, K. J., Tavares, A. A. S., Mattiucci, D., Scheller, E. L., Papanastasiou, G., Gray, C., Sinton, M. C., Ramage, L. E., McDougald, W. A., Lovdel, A. et al. (2020). Bone marrow adipose tissue is a unique adipose subtype with distinct roles in glucose homeostasis. *Nat. Commun.* 11, 3097. 10.1038/s41467-020-16878-2 32555194 PMC7303125

[BIO062396C30] Tavassoli, M. (1976). Ultrastructural development of bone marrow adipose cell. *Acta Anat (Basel)* 94, 65-77. 10.1159/000144545 961340

[BIO062396C31] Xu, W., Mesa-Eguiagaray, I., Morris, D. M., Wang, C., Gray, C. D., Sjöström, S., Papanastasiou, G., Badr, S., Paccou, J., Li, X. et al. (2025). Deep learning and genome-wide association meta-analyses of bone marrow adiposity in the UK Biobank. *Nat. Commun.* 16, 99. 10.1038/s41467-024-55422-4 39747859 PMC11697225

[BIO062396C32] Yeung, D. K., Griffith, J. F., Antonio, G. E., Lee, F. K., Woo, J. and Leung, P. C. (2005). Osteoporosis is associated with increased marrow fat content and decreased marrow fat unsaturation: a proton MR spectroscopy study. *J. Magn. Reson. Imaging* 22, 279-285. 10.1002/jmri.20367 16028245

[BIO062396C33] Zhong, L., Yao, L., Tower, R. J., Wei, Y., Miao, Z., Park, J., Shrestha, R., Wang, L., Yu, W., Holdreith, N. et al. (2020). Single cell transcriptomics identifies a unique adipose lineage cell population that regulates bone marrow environment. *eLife* 9, e54695. 10.7554/eLife.54695 32286228 PMC7220380

[BIO062396C34] Ziegenhain, C., Vieth, B., Parekh, S., Reinius, B., Guillaumet-Adkins, A., Smets, M., Leonhardt, H., Heyn, H., Hellmann, I. and Enard, W. (2017). Comparative analysis of single-cell RNA sequencing methods. *Mol. Cell* 65, 631-643.e4. 10.1016/j.molcel.2017.01.023 28212749

